# Seed dispersal by wind decreases when plants are water‐stressed, potentially counteracting species coexistence and niche evolution

**DOI:** 10.1002/ece3.8305

**Published:** 2021-11-04

**Authors:** Jinlei Zhu, Nataša Lukić, Verena Rajtschan, Julia Walter, Frank M. Schurr

**Affiliations:** ^1^ Institute of Landscape and Plant Ecology University of Hohenheim Stuttgart Germany; ^2^ Institute of Soil Science and Land Evaluation University of Hohenheim Stuttgart Germany; ^3^ Institute of Physics and Meteorology University of Hohenheim Stuttgart Germany; ^4^ LTZ Augustenberg Rheinstetten Germany

**Keywords:** Anemochory, community dynamics, context‐dependent seed dispersal, drought, source‐sink dynamics, water logging

## Abstract

Hydrology is a major environmental factor determining plant fitness, and hydrological niche segregation (HNS) has been widely used to explain species coexistence. Nevertheless, the distribution of plant species along hydrological gradients does not only depend on their hydrological niches but also depend on their seed dispersal, with dispersal either weakening or reinforcing the effects of HNS on coexistence. However, it is poorly understood how seed dispersal responds to hydrological conditions. To close this gap, we conducted a common‐garden experiment exposing five wind‐dispersed plant species (*Bellis perennis*, *Chenopodium album*, *Crepis sancta*, *Hypochaeris glabra*, and *Hypochaeris radicata*) to different hydrological conditions. We quantified the effects of hydrological conditions on seed production and dispersal traits, and simulated seed dispersal distances with a mechanistic dispersal model. We found species‐specific responses of seed production, seed dispersal traits, and predicted dispersal distances to hydrological conditions. Despite these species‐specific responses, there was a general positive relationship between seed production and dispersal distance: Plants growing in favorable hydrological conditions not only produce more seeds but also disperse them over longer distances. This arises mostly because plants growing in favorable environments grow taller and thus disperse their seeds over longer distances. We postulate that the positive relationship between seed production and dispersal may reduce the concentration of each species to the environments favorable for it, thus counteracting species coexistence. Moreover, the resulting asymmetrical gene flow from favorable to stressful habitats may slow down the microevolution of hydrological niches, causing evolutionary niche conservatism. Accounting for context‐dependent seed dispersal should thus improve ecological and evolutionary models for the spatial dynamics of plant populations and communities.

## Graphical abstract

To test how seed dispersal by wind responds to abiotic contexts, we estimate fecundity and seed dispersal of five plant species under various hydrological conditions by combining common‐garden experiments and mechanistic modeling, and quantified the relationship between seed production and seed dispersal. There was a general positive relationship between seed production and dispersal distance. The positive relationship between seed production and dispersal may reduce the concentration of each species to the environments favorable for it, thus counteracting species coexistence.

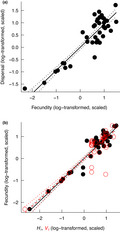

## 1 INTRODUCTION

Niche theory plays a central role in explaining species–environment relationships (Chase & Leibold, 2003; Hutchinson, 1961; Schurr et al., 2012). Hydrological niche segregation (HNS), in particular, has been widely adopted to explain species coexistence of plants (Silvertown, 2004; Silvertown et al., 1999, 2015). HNS causes plant species to coexist due to partitioning of space on fine‐scale soil‐moisture gradients (Araya et al., 2011), of water as a resource, and/or of temporally variable recruitment opportunities. HNS has been used to predict the relationship between functional traits and community structure (Herberich et al., 2017) and has received strong empirical support in various ecosystems (Brum et al., 2019; Palacio et al., 2017; Silvertown et al., 2015). For example, soil moisture influences species distributions in forests (Francis et al., 2020; McLaughlin et al., 2020) and grasslands (Bartelheimer & Poschlod, 2016; Moeslund et al., 2013). Despite strong empirical support, identifying the precise mechanisms generating HNS and its consequences is an ongoing challenge (Araya et al., 2011; Silvertown et al., 1999). Recent studies showed that germination (James et al., 2020) and seedling growth (Silvertown et al., 2012) can vary along hydrological gradients, suggesting possible underlying mechanisms. Ultimately, hydrological niches are determined by how local population growth (the balance of reproduction and mortality) responds to hydrological variation (e.g., Holt, 2009). Understanding HNS thus requires us to quantify how reproduction and mortality (and components thereof, such as seed production) respond to local hydrological conditions.

The coexistence‐promoting effects of small‐scale HNS can be reinforced or counteracted by seed dispersal (Hart et al., 2017; Levine & Murrell, 2003). In fact, dispersal is a central component of “growth‐density covariance” as a key coexistence mechanism in spatially heterogeneous environments (Barabás et al., 2018; Chesson, 2000; Hart et al., 2017). Positive covariance between intrinsic population growth rate and population density occurs if dispersal is generally limited or if context dependence reduces dispersal from favorable environments. Consequently, individuals in favorable environments experience stronger intraspecific competition which reinforces spatial storage effects and spatial coexistence of species (Hart et al., 2017). Conversely, if plants in favorable environments have increased seed dispersal, growth‐density covariance and the spatial storage effect will be weakened and species coexistence will be less likely. Hence, linking context‐dependent seed dispersal with HNS could help to understand the mechanisms of species coexistence in variable environmental conditions (Travis et al., 2013).

Context‐dependent seed dispersal may also play important roles in gene flow and the microevolution of hydrological niches. Evolutionary theory predicts selection for reduced seed dispersal in favorable habitats, leading to restricted gene flow (Levin et al., 2003; Ronce, 2007), which allows local adaptation of populations in unfavorable habitats (Savolainen et al., 2007). Reduced dispersal under favorable conditions could thus promote microevolution and differentiation of hydrological niches. Conversely, increased dispersal under favorable conditions can lead to asymmetrical gene flow from favorable to unfavorable habitats, which prevents local adaptation to unfavorable conditions (Aguilée et al., 2016; Kirkpatrick & Barton, 1997) and ultimately causes evolutionary niche conservatism (Wiens & Graham, 2005).

There is growing realization that seed dispersal is context‐dependent, because dispersal traits depend on the environment external to the individual (Beckman et al., 2019; Clobert et al., 2012). However, our understanding of the context dependence of seed dispersal is impeded by a lack of studies (Rogers et al., 2019). An exception is the study of Teller et al. (2014) who showed that the dispersal ability of *Carduus nutans* seeds decreased in drought‐stressed compared with well‐watered conditions. Yet, it is still unclear whether this response of seed dispersal to drought stress is a general trend, and how seed dispersal responds to varying hydrological conditions including stress by both drought and waterlogging. Addressing these questions requires a mechanistic analysis of seed dispersal.

Seed dispersal by wind is one of the most important seed dispersal mechanisms in the plant kingdom (Willson et al., 1990), and by far the best studied dispersal mechanism (Nathan et al., 2011). Wind dispersal depends on dispersal traits and the dispersal environment. Crucial dispersal traits are seed terminal velocity (the constant velocity a falling seed reaches in the air, *V_t_*) and seed release height (*H_r_*) (Katul et al., 2005; Nathan et al., 2001, 2011; Soons et al., 2004; Tackenberg, 2003). Key properties of the physical dispersal environment are wind conditions such as mean horizontal wind speed, vertical uplift, and turbulence (Katul et al., 2005; Nathan et al., 2001, 2011). Significant progress has been made in quantifying the physical dispersal environment at various scales. Global wind data are either directly accessible (Fick & Hijmans, 2017) or can be simulated (Kling & Ackerly, 2020). On fine scales, high‐resolution three‐dimensional wind data can be either directly measured *in situ* with advanced remote sensing systems (Wulfmeyer et al., 2018) or can be simulated with mechanistic microclimatic models (Maclean, 2020). In contrast, dispersal traits vary with biotic and abiotic contexts and are more challenging to predict. Therefore, the key to quantifying context‐dependent seed dispersal by wind is to determine how dispersal traits respond to environmental contexts.

In this study, we explore how seed dispersal by wind responds to hydrological conditions. Specifically, we test three hypotheses: (1) HNS causes species to differ in how hydrological conditions affect seed production, (2) hydrological conditions affect dispersal traits and seed dispersal distance, (3) hydrological conditions that increase fecundity also increase per‐seed dispersal distance, thereby weakening the effect of HNS on species coexistence. A positive relationship between fecundity and seed dispersal would reduce growth‐density covariance and could thus counteract the spatial storage effect by which HNS promotes coexistence.

## 2 MATERIAL AND METHODS

### 2.1 Experimental design

We carried out a common‐garden experiment in 2019 at the Heidfeldhof research station (48.7126°N, 9.1900°E, 396 m a.s.l), University of Hohenheim, Germany. We used the widely distributed ruderal plant species *Chenopodium album* L. (*Amaranthaceae*), *Crepis sancta* (L.) Bornm. (*Asteraceae*), *Hypochaeris glabra* L. (*Asteraceae*), *Hypochaeris radicata* L. (*Asteraceae*), and *Bellis perennis* L. (*Asteraceae*) as study species, covering a variety of seed morphologies (Table S1). These species commonly co‐occur in natural communities (Sabatini et al., 2021) and have different Ellenberg indicator values for moisture (*F*‐value; Ellenberg & Leuschner, 2010, Domina et al., 2018): *C. album*, *C. sancta*, *H. glabra*, *H. radicata*, and *B. perennis* with an *F*‐value of 4, 2, 3, 5, and 5, respectively, suggesting that they may occupy different hydrological niches. For each species, we transplanted two seedlings into the center area (diameter 10 cm) of each pot with a volume of 20 L (height 28 cm, diameter 35 cm) filled with sandy loam (14% clay, 70% sand, 16% silt; pH 7.88), and randomly put each group of seven pots into a pool with a volume of 275 L (height 35 cm, diameter 106 cm). We applied four hydrological treatments with different water table depth (WTD) below the soil surface in the pots: “dry” (WTD 28 cm), “mesic” (WTD 22 cm), “late pulsed waterlogging” (WTD 22 cm throughout the experiment except 2 weeks of treatment when the soil surface in the pots was flooded), which was to mimic a flooding event, and "waterlogged” (WTD 5 cm) (Lukić et al., 2020; Walter, 2020) (Figure S1). We used three replicates for each species in each treatment, except that the “late pulsed waterlogging” treatment was only applied to *C. sancta*, *H. glabra*, and *H. radicata*—species with pappus as seed appendage. In total, 54 pots were used in the experiment.

### 2.2 Plant survival, seed production, and dispersal traits

During the experiment, we monitored survival of both target and the neighboring plants in each pot and recorded the date when a plant died. For *C. sancta*, *H. glabra*, *H. radicata*, and *B. perennis*, we carefully collected ten seed heads (capitula) from each target plant when possible, stored each seed head separately to keep the seed appendages intact, and used these seed heads to determine seed production, *H_r_* and *V_t_*. Before collection, we recorded the vertical distance between soil surface and the seed head as *H_r_*. After collection, we air‐dried the seed heads in the laboratory for 2 weeks. We then carefully separated and counted the number of seeds from three seed heads that were collected from the lowest, median, and highest *H_r_*, calculated the mean seed number per‐seed head, and estimated the seed production by multiplying the mean seed number by the total number of seed heads. In *C. album*, inflorescence clusters are attached to the leaf axils and at terminus of stems, and each branch has the similar structure as the whole plant. Therefore, for *C. album*, we first randomly measured ten values of *H_r_* from each target plant, covering the distribution of seeds on the plant. We then sampled three 5‐cm‐long plant segments, air‐dried and weighed them, separated and counted the number of seeds in each segment, and estimated seed production by multiplying the mean seed number per gram of plant segment and the total weight of the target plant. For each target plant, we randomly sampled nine seeds and measured *V_t_* in three steps. First, we shot videos of descending seeds with a high‐speed camera (130 fps, acA1920‐155um, BASLER). Second, from the videos we extracted the seed's three‐dimensional coordinates over time with ImageJ (Schneider et al., 2012). Third, we fitted an acceleration model describing variation in vertical coordinates with time and calculated *V_t_*.

### 2.3 Dispersal environment

We determined mean horizontal wind speed, aerodynamic roughness length, and friction velocity with an eddy covariance station (Wulfmeyer et al., 2018), and fitted a logarithmic wind velocity profile to the wind data to calculate horizontal wind speed at any height of interest,

(1) $$u=\frac{u_{*}}{k}\ln\frac{z‐d}{z_{0}}$$

where *u* is mean horizontal wind speed at the height *z*; *u* ^*^ is the friction velocity; *k* is von Kάrmάn's constant (0.41); *d* is zero‐plane displacement distance; and *z*_0_ is aerodynamic roughness length (Monteith & Unsworth, 2013).

### 2.4 Simulation of seed dispersal

We used the WALD mechanistic model (Katul et al., 2005) to simulate seed dispersal,

(2) $$p\left(x\right)={\left(\frac{\lambda }{2\pi x^{3}}\right)}^{1/2}\exp\left[‐\frac{\lambda {\left(x‐\mu \right)}^{2}}{2\mu^{2}x}\right]$$

where *x* is predicted seed dispersal distance, $\mu =H_{r}U/V_{t}$, $\lambda ={\left(H_{r}/\sigma \right)}^{2}$, *U* is the horizontal wind speed, σ is a turbulent flow parameter reflecting wind speed variation (see detailed calculation in Supporting information). For each species under each hydrological condition, we simulated 10^5^ seed dispersal events (Supporting information). Each seed released was dispersed by randomly drawing a distance from Eq. (2) with a unique set of parameters for *U*, *H_r_*, *V_t_*, and *σ* following Travis et al. (2011) and Schurr (2013). *U* was randomly drawn from a Weibull distribution fitted to the wind data. *H_r_* and *V_t_* were simulated from the linear mixed‐effects models that respectively quantified the relationship between variation in *H_r_* and *V_t_* and hydrological conditions and species identity.

### 2.5 Data analyses

We determined the relationships between plant survival, seed production, fitness proxy, dispersal traits, seed dispersal distance, and hydrological conditions using generalized linear mixed‐effects models (GLMMs) and linear mixed‐effects models (LMMs) (*lme4* package; Bates et al., 2015) in R version 4.0.3 (R Core Team, 2020). Plant survival was analyzed with a GLMM in which survival was the response variable, hydrological conditions, species identity, and their interaction were fixed‐effect explanatory variables, and pot identity nested within pool identity was included as random effect, with the binomial error distribution. We used the product of survival of each plant species and reproductive output for each individual as a fitness proxy for each focal plant under each treatment, following Sheppard and Schurr (2019). In the LMMs log‐transformed seed production (adding one to make sure that zero values remain zero after transformation), fitness proxy, seed release height (*H_r_*), seed terminal velocity (*V_t_*), and dispersal distance (*x*) were the response variables; hydrological conditions, species identity and their interaction were fixed‐effect explanatory variables; and pot identity nested within pool identity was included as random effect. For analysis of *V_t_*, we additionally included a random effect of seed head (nested within pot and pool identity). For the multi‐species analysis of the seed production–dispersal relationship, we accounted for the large interspecific variation in seed production and seed dispersal by log‐transforming and scaling mean seed production and dispersal distance of each target plant within each species under each watering treatment. We determined the relationship between seed dispersal distance and seed production using linear regression, in which log‐transformed and scaled mean seed dispersal distance was the response variable, log‐transformed and scaled mean seed production, species identity, and their interaction were explanatory variables. We then used similar linear regressions to quantify the contribution of *H_r_* and *V_t_* to seed production, in which log‐transformed and scaled mean seed production was the response variable, log‐transformed and scaled mean *H_r_* and log‐transformed and scaled mean *V_t_*, respectively, and their interaction with species were explanatory variables.

Data on seed production, seed dispersal traits, simulated dispersal distances, survival, and fitness proxy are openly available on the Dryad Digital Repository (Zhu et al., 2022).

## 3 RESULTS

### 3.1 Effects of hydrological conditions on plant survival, seed production, and fitness proxy

**FIGURE 1:**
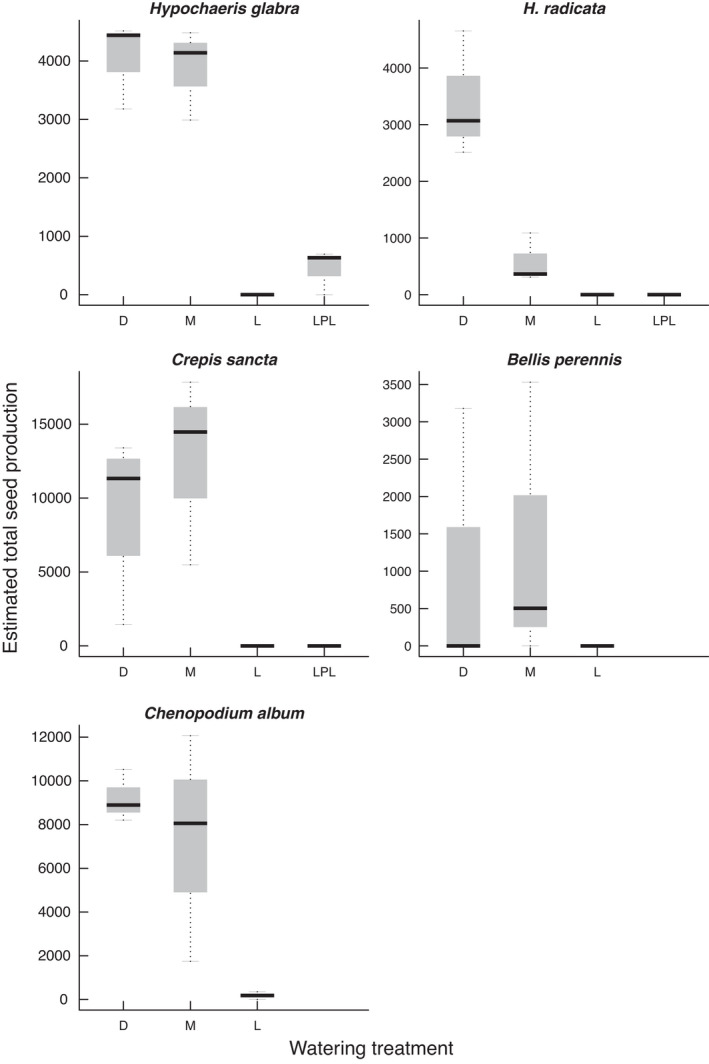
Effects of watering regime on seed production. Watering treatments are dry (D), mesic (M), waterlogged (L), and late pulsed waterlogging (LPL)

Species differed in the response of plant survival, seed production, and fitness proxy to hydrological conditions. Plant survival depended on the interaction between species and hydrological conditions (likelihood‐ratio test, $\chi_{10df}^{2}=18.67$, *p* = .045). Seed production also depended on the interaction between species and hydrological conditions ($\chi_{10df}^{2}=23.44$, *p* = .009). Although all species produced fewest seeds in the waterlogged treatment, they did not have the same optimum—*B. perennis* and *C. sancta* produced most seeds in the mesic treatment, whereas *H. glabra*, *H. radicata*, and *C. album* reached optimum seed production in the dry condition (Figure 1). Moreover, fitness proxy depended on the interaction between species and hydrological conditions ($\chi_{10df}^{2}=27.92$, *p* = .002) (Figure S2).

### 3.2 Effects of hydrological conditions on dispersal traits

**FIGURE 2:**
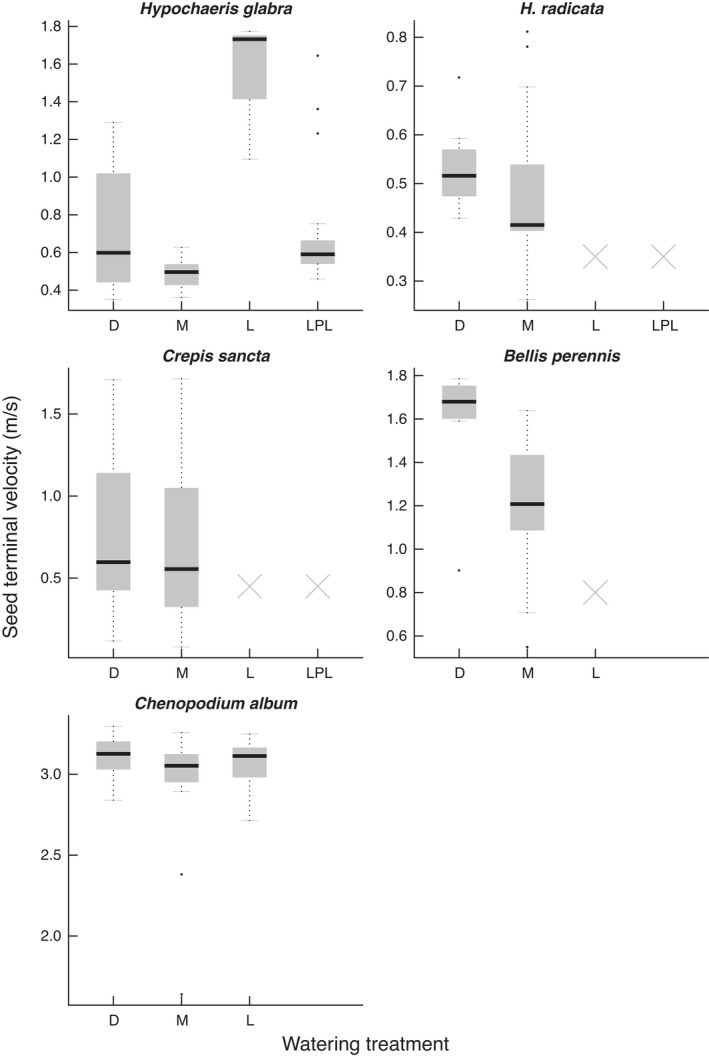
Effects of watering regime on seed release height. Watering treatments are dry (D), mesic (M), waterlogged (L), and late pulsed waterlogging (LPL). Crosses indicate that the plants in the respective treatment did not produce seeds

**FIGURE 3:**
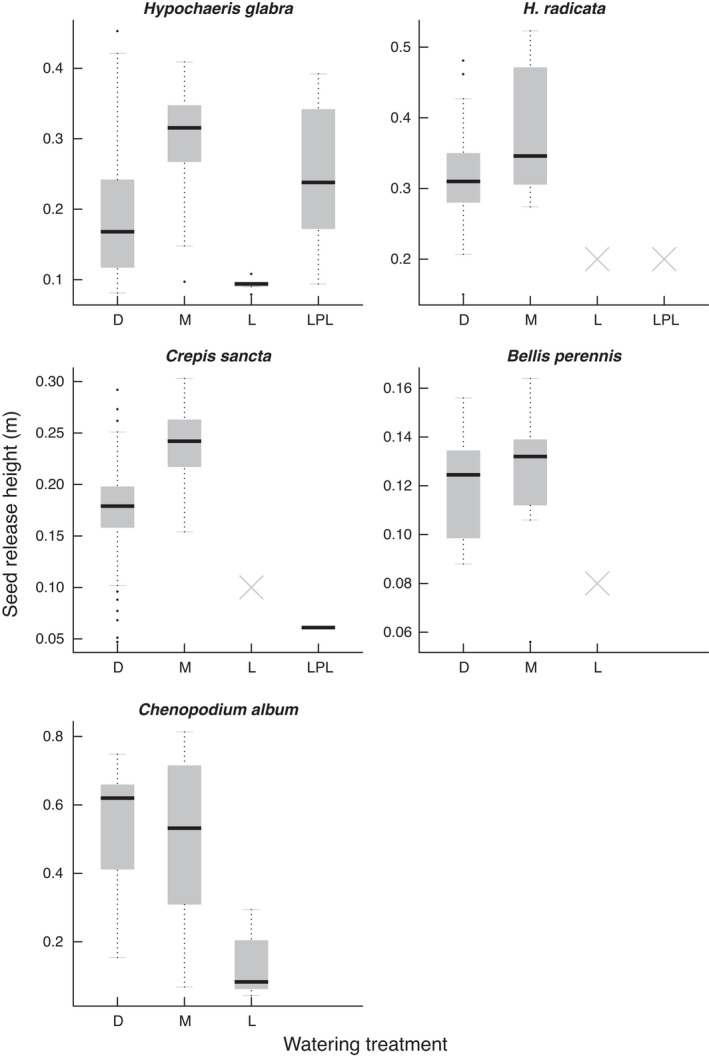
Effects of watering regime on seed terminal velocity. Watering treatments are dry (D), mesic (M), waterlogged (L), and late pulsed waterlogging (LPL). Crosses indicate that the plants in the respective treatment did not produce seeds

Seed release height (*H_r_*) did not depend on the interaction between species and hydrological conditions ($\chi_{6df}^{2}=10.52$, *p* = .104) but on the additive effects of both species ($\chi_{4df}^{2}=32.39$, *p* < .001) and hydrological conditions ($\chi_{3df}^{2}=28.67$, *p* < .001). *H_r_* was lowest in the waterlogged condition and was highest in the mesic condition in four out of five species except *C. album*, which had the highest *H_r_* in the dry treatment (Figure 2). In contrast, seed terminal velocity (*V_t_*) depended on the interaction between species and hydrological conditions ($\chi_{5df}^{2}=15.79$, *p* = .007). *V_t_* was lowest in the mesic condition and highest in the dry treatment in four out of five species except *H. glabra*, which had the highest *V_t_* in the waterlogged condition (Figure 3).

### 3.3 Effects of hydrological conditions on predicted seed dispersal distance

**FIGURE 4:**
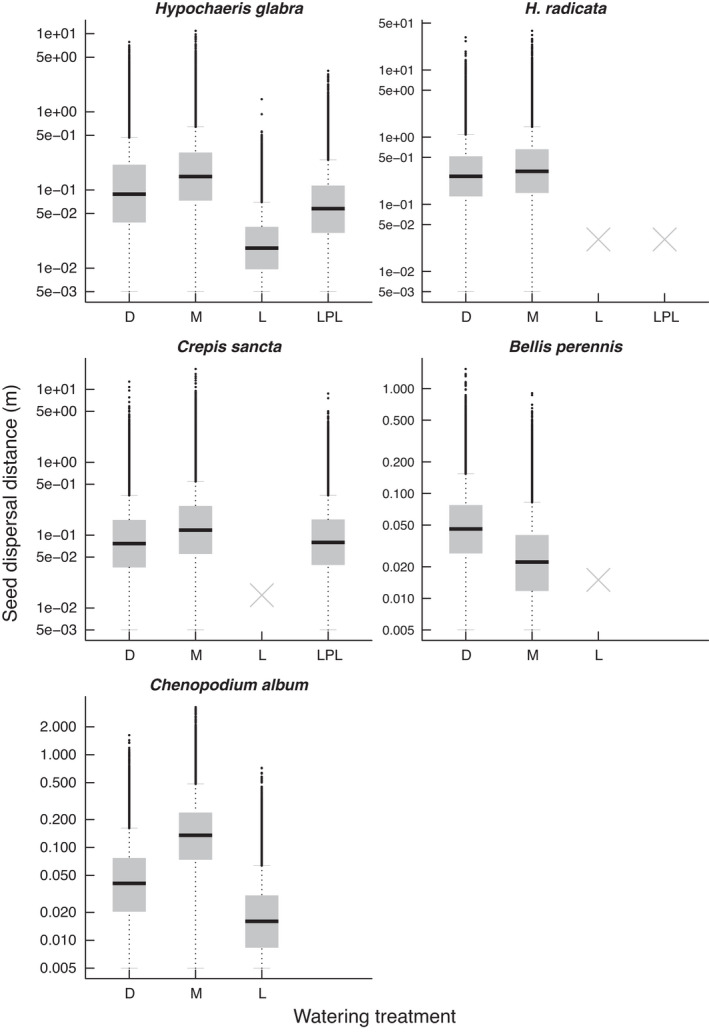
Effects of watering regime on predicted seed dispersal distance. Watering treatments are dry (D), mesic (M), waterlogged (L), and late pulsed waterlogging (LPL). Crosses indicate that the plants in the respective treatment did not produce seeds. Note that the y‐axis was plotted on a logarithmic scale

Predicted mean seed dispersal distances did not depend on an interaction between species and hydrological conditions ($\chi_{6df}^{2}=2.807$, *p* = .833), but on the additive effects of both species ($\chi_{4df}^{2}=30.996$, *p* < .001) and hydrological conditions ($\chi_{3df}^{2}=8.140$, *p* = .043). Mean predicted dispersal distances were longest in the mesic condition for four out of five species except *H. glabra*, which had the longest mean dispersal distance in the dry treatment. Mean dispersal distance was shortest in the waterlogged treatment for *H. glabra* and *C. album*, but in the dry treatment for *H. radicata*, *C. sancta*, and *B. perennis* (Figure 4). There was significant variation in dispersal distance among treatments. For instance, mean dispersal distance was 14‐fold (*H. glabra*) and 9‐fold (*C. album*) higher in the mesic treatment than that in the waterlogged treatment.

### 3.4 Relationship between seed dispersal and seed production

**FIGURE 5:**
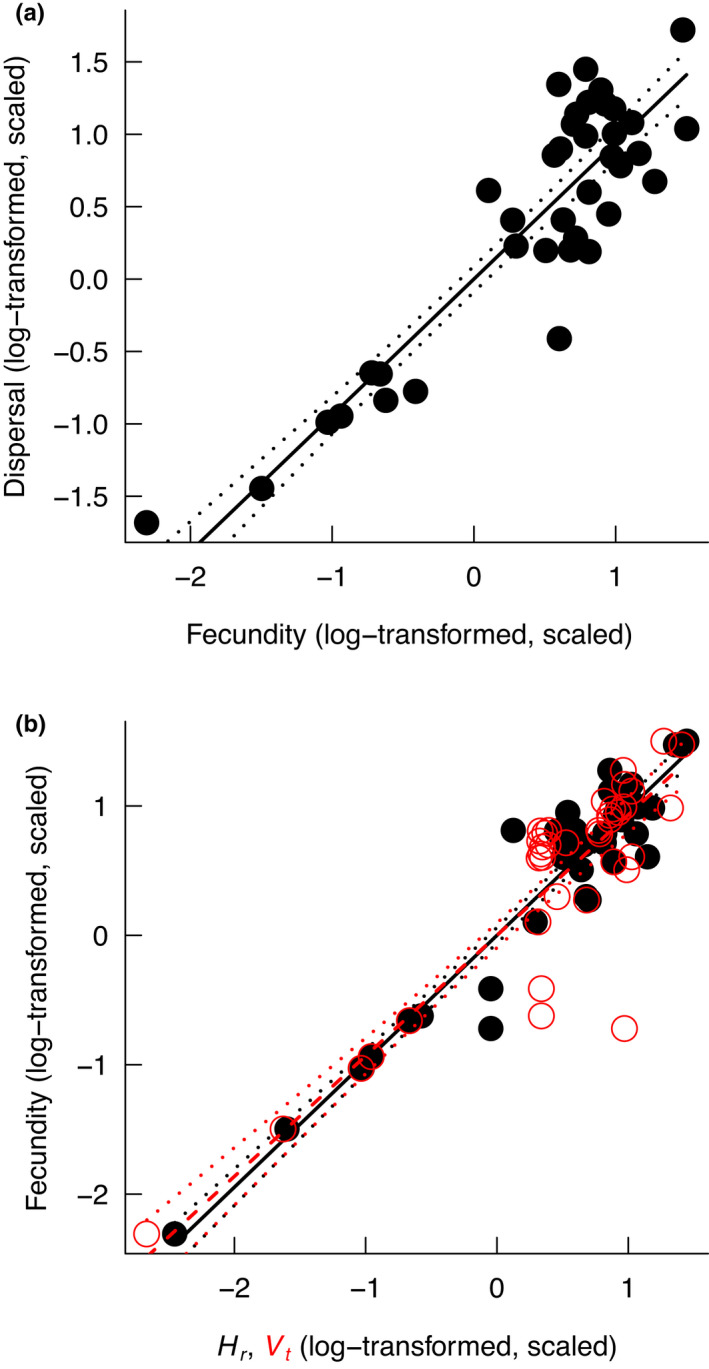
(a) Relationship between dispersal distance and fecundity. (b) Relationship between fecundity and seed release height (solid points and solid line) and seed terminal velocity (red empty points and dashed line). Solid and dashed lines indicate model predictions, and dotted lines indicate 95% confidence intervals

We found a positive relationship between mean dispersal distance and seed production across all the study species ($F_{1,53}=407.8$, *p* < .001) (Figure 5a). This positive relationship between dispersal and seed production arose because plants with higher seed production had higher *H_r_* (Figure 5b; *R* ^2^ = .95) and—to a lesser extent—because they had higher *V_t_* (Figure 5b; *R* ^2^ = .86).

## 4 DISCUSSION

The five study species showed species‐specific responses of seed production to hydrological conditions (Figure 1), confirming their hydrological niche segregation (HNS). We also found species‐specific responses of dispersal traits and predicted seed dispersal distances to hydrological conditions (Figures 2, 3, 4). Despite these species‐specific responses, we found a generally positive relationship between seed dispersal and seed production (Figure 5a). When growing in stressful hydrological conditions, plants not only produce fewer seeds, but they may also have lower *H_r_* (Figure 5b; Figure S3a) and greater *V_t_* (Figure 5b; Figure S3b), causing their seeds to be dispersed over shorter distances. In the following, we discuss potential mechanisms underlying this context‐dependent seed dispersal, how context‐dependent dispersal may modify the effect of HNS on species coexistence, and how it may affect the evolution of hydrological niches.

### 4.1 Mechanisms of context‐dependent seed dispersal by wind

Context‐dependent wind dispersal arises from the species‐specific responses of dispersal traits (*H_r_* and *V_t_*) to hydrological conditions. Lower *V_t_* increases the flying time of seeds, and higher *H_r_* increases both flying time and the wind speed that flying seeds experience, leading to longer dispersal distances (Soons et al., 2004). In stressful hydrological environments, plants grow shorter and tend to produce fewer but larger seeds (with higher *V_t_*), causing shorter dispersal distances. Additionally, seed production increases more strongly with *H_r_* than it decreases with *V_t_*. The correlated response of seed production and *H_r_* to hydrological stress causes wind‐driven seed dispersal to be highest in favorable environments and lowest in stressful environments. In fact, we posit that the positive relationship between seed production and context‐dependent seed dispersal also holds for dispersal mechanisms other than wind. This is because stress generally reduces both seed production and plant height and because plant height is positively correlated with seed dispersal distance across different dispersal mechanisms (Thomson et al., 2011).

### 4.2 The relevance of context‐dependent seed dispersal

Context‐dependent seed dispersal can have important ecological and evolutionary consequences (Clobert et al., 2012). By failing to consider the context dependence of seed dispersal, one could overestimate the dispersal ability of plants under drought‐stressed conditions and thereby underestimate the probability of local extinction (Teller et al., 2015). Additionally, our study shows two other consequences of failing to consider the context dependence of seed dispersal: One could underestimate the extinction probability of plants in waterlogged conditions and underestimate the dispersal ability of plants in favorable hydrological conditions, thereby underestimating spread rates (which should be particularly relevant for invasive species). Hence, quantifying context‐dependent seed dispersal directly leads to more realistic predictions of dispersal distances, which are valuable for conservation planning and plant invasion control (Trakhtenbrot et al., 2005).

Although this study focused on seed dispersal at small spatial scales, context‐dependent seed dispersal might also be relevant at large spatial scales. For instance, the environments at leading and trailing edges of species’ ranges could differ substantially from the range core (Thuiller et al., 2008), and context‐dependent seed dispersal may have different effects on leading and trailing edges and produce complex eco‐evolutionary dynamics (Nadeau & Urban, 2019; Weiss‐Lehman & Shaw, 2020). Increased dispersal in favorable environments also means that niche‐distribution mismatches resulting from migration limitation (Pagel et al., 2020) might be strongest in stressful conditions where plants have reduced dispersal, whereas these mismatches should be reduced in favorable conditions. It is thus worth exploring whether the incorporation of context‐dependent seed dispersal into dynamic range models could improve predictions of geographic distributions of plant species under changing climate (Pagel & Schurr, 2012; Schurr et al., 2012).

### 4.3 Context‐dependent seed dispersal and hydrological niche segregation

There can be a bidirectional interaction between context‐dependent seed dispersal and HNS. Our findings confirm that hydrological conditions can alter dispersal traits, leading to intraspecific variation in dispersal ability. The detected increased seed dispersal under favorable hydrological conditions could reduce growth‐density covariance, thereby reducing intraspecific competition and increasing interspecific competition between species with different hydrological niche optima (Hart et al., 2017). This reduced growth‐density covariance should counteract the spatial storage effect by which HNS promotes coexistence (Hart et al., 2017). Importantly, however, HNS is not invalidated if seed dispersal is generally higher under favorable hydrological conditions, and this is because the resulting source‐sink dynamics cause mismatches between niches and distributions (Pagel et al., 2020; Schurr et al., 2012). Consequently, niche segregation should in this case be even stronger than suggested by observed species sorting along hydrological gradients (Araya et al., 2011; Silvertown et al., 1999).

### 4.4 Context‐dependent seed dispersal and niche evolution

Context‐dependent seed dispersal affects gene flow and may influence local adaptation. The detected context‐dependent seed dispersal might cause unidirectional dispersal from optimal to suboptimal habitats, resulting in “migration load” that slows down the local adaptation of lineages to suboptimal habitats (Kirkpatrick & Barton, 1997; Savolainen et al., 2007). Consequently, increased dispersal from optimal habitats may reduce intraspecific niche differentiation (Schiffers et al., 2014) and ultimately contribute to evolutionary niche conservatism (Wiens et al., 2010).

### 4.5 Conclusions

Our finding that seed dispersal by wind decreases when plants are hydrologically stressed, provides concrete evidence that seed dispersal is context‐dependent, highlighting consequences of hydrological niche segregation for fine‐scale dispersal. The detected increase in dispersal in favorable environments might counteract the effects of HNS on species coexistence and could potentially slow down the microevolution of hydrological niches, causing evolutionary niche conservatism. Hence, our findings may offer new insights into mechanisms linking seed dispersal to species coexistence and niche evolution. Moreover, accounting for context‐dependent seed dispersal should improve models for the spatial dynamics of plant populations and communities.

## CONFLICT OF INTEREST

The authors have no conflict of interest.

## AUTHOR CONTRIBUTIONS

**Jinlei Zhu:** Conceptualization (lead); Data curation (lead); Formal analysis (lead); Investigation (lead); Methodology (lead); Project administration (lead); Resources (lead); Software (lead); Visualization (lead); Writing‐original draft (lead); Writing‐review & editing (lead). **Nataša Lukić:** Conceptualization (supporting); Data curation (supporting); Investigation (supporting); Methodology (supporting); Project administration (supporting); Writing‐original draft (supporting); Writing‐review & editing (supporting). **Verena Rajtschan:** Data curation (supporting); Investigation (supporting); Methodology (supporting); Project administration (supporting); Resources (supporting); Writing‐original draft (supporting). **Julia Walter:** Conceptualization (supporting); Funding acquisition (lead); Methodology (supporting); Project administration (supporting); Writing‐original draft (supporting); Writing‐review & editing (supporting). **Frank M. Schurr:** Conceptualization (supporting); Data curation (supporting); Formal analysis (supporting); Funding acquisition (supporting); Methodology (supporting); Project administration (supporting); Resources (supporting); Software (supporting); Supervision (lead); Visualization (supporting); Writing‐original draft (supporting); Writing‐review & editing (supporting).

## Supporting information

Supplementary Material

## Data Availability

Data on seed production, seed dispersal traits, simulated dispersal distances, survival, and fitness proxy are openly available from the Dryad Digital Repository (Zhu et al., 2022).
