# Supplementary material for: Seed dispersal by wind decreases when plants are water‐stressed, potentially counteracting species coexistence and niche evolution
Source: Ecol Evol. 2021 Nov 4;11(22):16239–49. doi: 10.1002/ece3.8305 (PMC8601872; doi:10.1002/ece3.8305)
Supplement: Supplementary file 1 — Supplementary Material [file ECE3-11-16239-s001.docx]

Supporting information for

**Seed dispersal by wind decreases when plants are water-stressed, potentially counteracting species coexistence and niche evolution**

**Fig.S1.** Schematic diagram of the experimental design. Seven pots, in each of which two seedlings of a study species were transplanted, were randomly put into a pool in which water table was controlled by drilling holes at a specific height at the outside wall of the pool.

**Fig. S2.** Effects of watering regime on fitness proxy. Watering treatments are dry (D), mesic (M), waterlogged (L), and late pulsed waterlogging (LPL).

**Fig. S3.** Effects of stressful and favourable hydrological conditions on (a) seed release height and (b) seed terminal velocity. Favourable conditions were the hydrological conditions in which target plants had the highest seed production, and stressful conditions were other hydrological conditions than favourable conditions in the experiment (details see Material and Methods and Results in the main text).

**Table S1.** Information of study species

| Species | Height (cm) | Diaspore morphology | Diaspore appendages | World distribution | Habitat | Flowering period | Life form |
| --- | --- | --- | --- | --- | --- | --- | --- |
| *Chenopodium album* | 30-150 | Pericarp adnate to seed, 1.2-1.5 mm in diameter | None | Cosmopolitan | Roadsides, fields, gardens, waste places | 5-10 | Therophyte |
| *Crepis sancta* | 5-30 | Dimorphic, inner achenes 3-4 mm long, with pappus; outer achenes 3-5 mm long, without pappus | Pappus, 4-5 mm long | East Mediterranean, Asia-Temperate, Europe, Africa | Roadsides, vineyards, railroad areas, warm locations | 4-5 | Therophyte |
| *Hypochaeris glabra* | 10-30 | Dimorphic, inner achenes with beak 6-8 mm long, outer achenes without beak, 3-4 mm long | Pappus, inner 9.5-15 mm long; outer 3-4 mm long | Native to Asia-Temperate, Europe, Northern Africa | Roadsides, sandy fallow fields | 7-9 | Therophyte |
| *H. radicata* | 15-60 | Inner achenes 6-10 mm long; outer achenes 8-17 mm long | Pappus | Native to Europe and Northern Africa | Meadows | 6-10 | Perennial hemicryptophyte |
| *Bellis perennis* | 10-25 | Achenes 1 mm long | None | Native to Northern Africa, Southwestern Asia, and Europe | Meadows, pastures, lawns | 2-11 | Perennial hemicryptophyte |

# Simulation of dispersal distance with the WALD mechanistic model

The WALD model is an analytical mechanistic model derived from a simplified 3-D stochastic dispersion model that retains the essential physics contained within the more computationally intensive coupled Eulerian-Lagrangian closure (CELC) model (Nathan et al. 2002). The analytical model reduces to the following WALD (or inverse Gaussian) distribution (eqn 5b in Katul et al. 2005) that describes the probability density of dispersal distances x

$p\left( x \right)=\left( \frac{\lambda}{2\pi x^{3}} \right)^{1/2}exp\left[ -\frac{\lambda\left( x-\mu\right)^{2}}{2\mu^{2}x} \right]$ (1)

where $\mu$ and $\lambda$ are dispersal kernel parameters, and $\mu={H_{r}U}/{V_{t}}$, $\lambda=\left( {H_{r}}/\sigma\right)^{2}$, *H_r_* is seed release height, *U* is the horizontal wind speed, *V_t_* is seed terminal velocity, and σ is a turbulent flow parameter reflecting wind speed variation (Katul et al. 2005, Skarpaas and Shea 2007).

$\sigma^{2}= \frac{4{\sigma_{w}}^{4}}{C_{0}\varepsilon U}$ (2)

where $\sigma_{w}$ is the vertical turbulence, $C_{0}$ is Kolmogorov constant (3.125), and $\varepsilon$ is the dissipation rate. Estimating $\varepsilon$ is challenging within the canopy (Katul et al. 2005), but we are mainly concerned with turbulent flow above the canopy, so we can assume $\varepsilon= {{u_{*}}^{3}}/{K\left( z-d \right)}$ and the constant $A_{w}\equiv{\sigma_{w}}/{u_{*}}\approx1.3$, where *z* is height above ground, *d* is zero-plane displacement distance (Skarpaas and Shea 2007). From these assumptions and equation (2), we obtain

$\sigma=2{A_{w}}^{2}\sqrt{\frac{K\left( z-d \right)u_{*}}{C_{0}U}}$ (3)

In the WALD simulation, each seed released was dispersed by randomly drawing a distance from the probability distribution function generated by the WALD model with parameters for wind speed, *H_r_* and *V_t_*. Wind speed was drawn from a Weibull distribution determined by wind measurements with an eddy covariance station at the Heidfeldhof research station (Wulfmeyer et al. 2018), University of Hohenheim, Germany. *H_r_* and *V_t_* were simulated from the linear mixed-effects models that respectively quantified the relationship between variation in *H_r_* and *V_t_* and hydrological conditions and species identity (more details see *Data analyses* in the paper).

**References**

Katul, G. G., et al. 2005. Mechanistic analytical models for long-distance seed dispersal by wind. - Am Nat 166: 368-81.

Nathan, R., et al. 2002. Mechanisms of long-distance dispersal of seeds by wind. - Nature 418: 409-13.

Skarpaas, O. and Shea, K. 2007. Dispersal patterns, dispersal mechanisms, and invasion wave speeds for invasive thistles. - Am Nat 170: 421-30.

Wulfmeyer, V. et al. 2018. A new research approach for observing and characterizing land-atmosphere feedback. - B. Am. Meteorol. Soc. 99: 1639-1667.
